# Saving Time for Patient Care by Optimizing Physician Note Templates: A Pilot Study

**DOI:** 10.3389/fdgth.2021.772356

**Published:** 2022-01-13

**Authors:** Rana Alissa, Jennifer A. Hipp, Kendall Webb

**Affiliations:** ^1^Department of Pediatrics, University of Florida College of Medicine Jacksonville, Jacksonville, FL, United States; ^2^Department of Medical Informatics, University of Florida College of Medicine Jacksonville, Jacksonville, FL, United States

**Keywords:** optimizing physician note, efficiency, time saving, improve documentation, provider satisfaction

## Abstract

**Background:** At times, electronic medical records (EMRs) have proven to be less than optimal, causing longer hours behind computers, shorter time with patients, suboptimal patient safety, provider dissatisfaction, and physician burnout. These concerning healthcare issues can be positively affected by optimizing EMR usability, which in turn would lead to substantial benefits to healthcare professionals such as increased healthcare professional productivity, efficiency, quality, and accuracy. Documentation issues, such as non-standardization of physician note templates and tedious, time-consuming notes in our mother-baby unit (MBU), were discussed during meetings with stakeholders in the MBU and our hospital's EMR analysts.

**Objective:** The objective of this study was to assess physician note optimization on saving time for patient care and improving provider satisfaction.

**Methods:** This quality improvement pilot investigation was conducted in our MBU where four note templates were optimized: History and Physical (H and P), Progress Note (PN), Discharge Summary (DCS), and Hand-Off List (HOL). Free text elements documented elsewhere in the EMR (e.g., delivery information, maternal data, lab result, etc.) were identified and replaced with dynamic links that automatically populate the note with these data. Discrete data pick lists replaced necessary elements that were previously free texts. The new note templates were given new names for ease of accessibility. Ten randomly chosen pediatric residents completed both the old and new note templates for the same control newborn encounter during a period of one year. Time spent and number of actions taken (clicks, keystrokes, transitions, and mouse-keyboard switches) to complete these notes were recorded. Surveys were sent to MBU providers regarding overall satisfaction with the new note templates.

**Results:** The ten residents' average time saved was 23 min per infant. Reflecting this saved time on the number of infants admitted to our MBU between January 2016 and September, 2019 which was 9373 infants; resulted in 2.6 hours saved per day, knowing that every infant averages two days length of stay. The new note templates required 69 fewer actions taken than the old ones (H and P: 11, PN: 8, DCS: 18, HOL: 32). The provider surveys were consistent with improved provider satisfaction.

**Conclusion:** Optimizing physician notes saved time for patient care and improved physician satisfaction.

## Introduction

### Problem Description

Our hospital, like many others in the nation, adopted the Epic Systems software (Epic, Verona, WI, United States) for electronic medical record (EMR) documentation in 2012. The Notes in our Mother Baby Unit (MBU) were minimally updated from the initial system loaded templates. Each of our pediatric residents had his/her own template, which generates numerous note arrangements. This lack of standardization led to confusion and dissatisfaction from the staff and community pediatricians who follow our newborns after they were discharged from our hospital.

### Available Knowledge

The introduction of EMR changed the format of health records and improved healthcare (1). It is essential to promote EMR interoperability in order to encourage eligible providers and hospitals to adopt and successfully demonstrate the meaningful use of certified EMR technology (2). However, with increased adoption of EMR systems, there is a possibility that issues related to the negative impact of increased and split cognitive workload on healthcare providers may occur (3). It has been affirmed over the years that well-designed interfaces improve efficiency; on the other hand, poorly designed interfaces steal minutes from busy schedules and increase human errors (3, 4). Healthcare providers are generally dissatisfied with EMR as it increases workload and stress while decreasing physician productivity and only compiles to physician burnout around the world (5). Also, the use of information communication technology (ICT) and its demand have been associated with higher provider stress and burnout (6). In 2017, Guo et al. aimed to find an association between “click burden” and physician frustration. The team applied in their study an EMR innovation technique, which resulted in less time spent on the computer and more time with patients (7).

### Rationale

The majority of the newborn medical record documentation is derived from the maternal medical condition reflected and is usually pulled from the mother's obstetrical prenatal and delivery documentation (8). The involvement of more than one department in the care of a newborn is complex. This specific patient care involves an intra-organizational communication boundary, which, if poor, can result in negative impact on patient care especially for providers who have high cognitive loads (9). This multidisciplinary team who takes care of the mother-infant couplet may have completely different documentation cultures (10). In order to improve productivity, communication, and quality of patient care, we embarked on this pilot project to optimize the physician note templates in our MBU in 2014. The project was also designed to enhance physician workflow, performance, efficiency, and satisfaction.

### Objective

The objective of this study was to assess physician note optimization on saving time for patient care and improving provider satisfaction.

## Materials and Methods

### Context

This project took place in the MBU at a regional perinatal center and a major academic university hospital, which is the largest in the Northern Florida and Southern Georgia region of the United States. Average infant deliveries are 2,800–3,200 per year.

**Figure 1:**
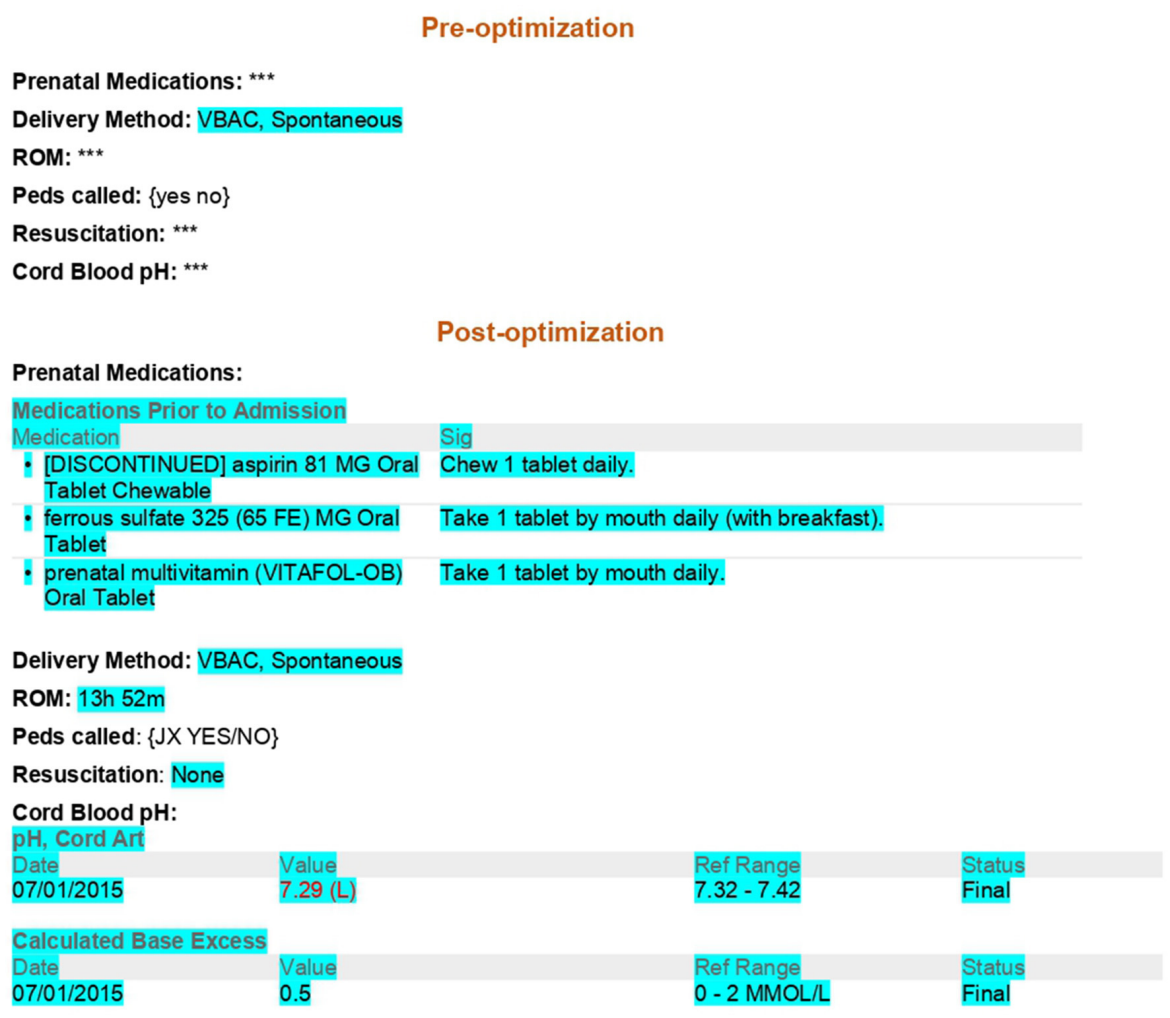
Maternal information comparison pre and post note optimization blue highlight: Auto generated data. ***: Manual entry of data required. {}: Pick list. Epic codes are omitted. VBAC, Vaginal birth after cesarean section; ROM, Rupture of membranes; Peds, Pediatrics.

**Figure 2:**
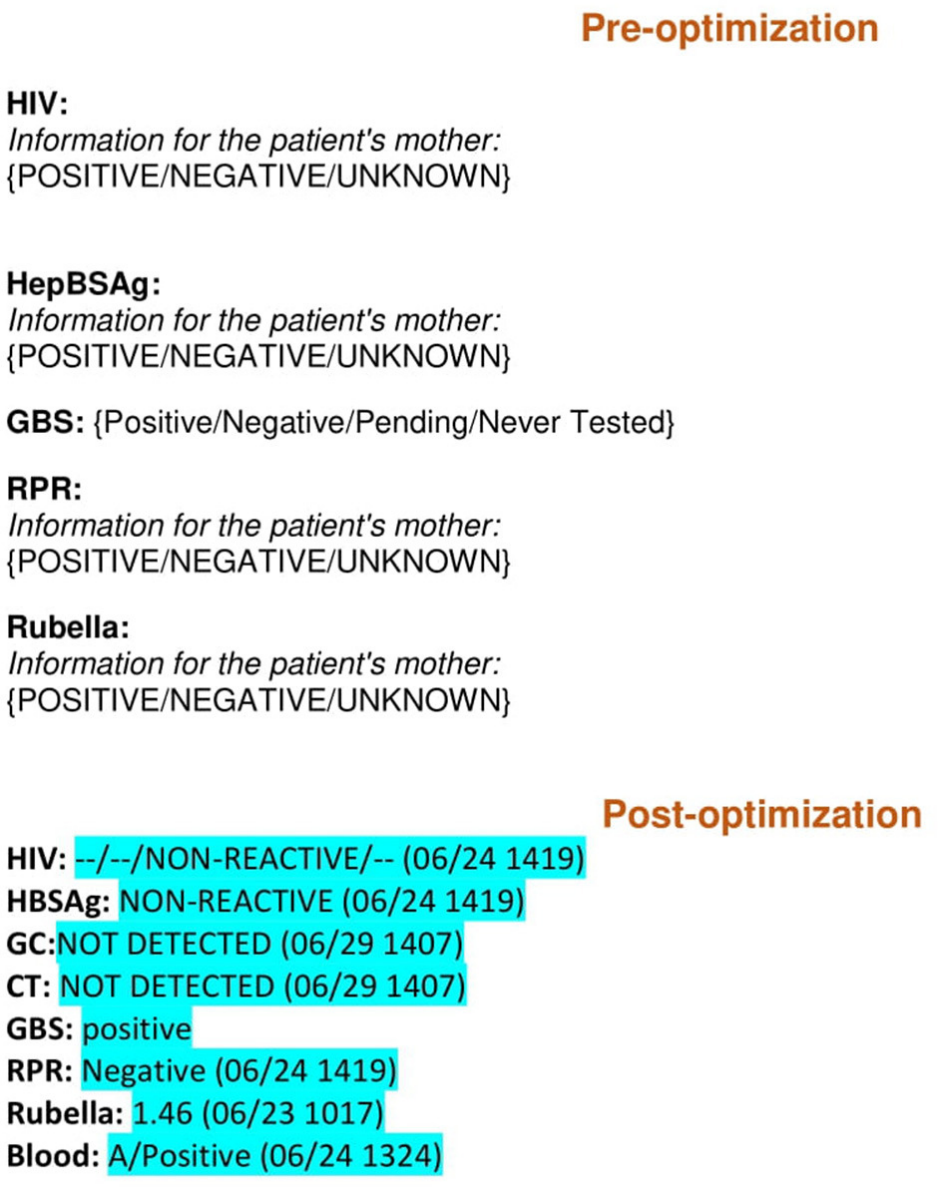
Maternal labs comparison pre and post note optimization blue highlight: Auto generated data. {}: Pick list. Epic codes are omitted. HIV, Human immunodeficiency virus; HBSAg, Hepatitis B antigen; GBS, Group B streptococcus; RPR, Rapid plasma reagin.

In 2014, documentation issues in the MBU unit, such as non-standardization of notes and amount of time spent creating them, were discussed and identified. This prompted meetings with stakeholders in the MBU and our hospital's EMR analysts to discuss their perspectives. The MBU stakeholders included pediatric faculty members, nurse practitioners who cover the MBU, and pediatric residents in their first and second year of training (Table 1). These stakeholders concluded *via* artifact analysis and observations that the majority of physician note data in the MBU was entered manually, such as maternal labs (at least seven required lab tests) (Figure 1), number of prenatal care visits, rupture of membrane duration (Figure 2), hearing test, bilirubin level, critical congenital heart disease (CCHD) screening results, and discharge weight. Percentage of weight change since birth was calculated on the day of discharge manually using a phone or a computer calculator. This value is very important for a newborn, because it reflects the effectiveness of feeding, voiding, and stooling in the infant. Also, the anticipatory guidance information provided to the new parents was added manually to the progress notes and discharge summaries (full examples are included in the Supplementary Material). After three meetings with this group, a formal project was planned and mapped out.

**Table 1:** Number of pediatric residents and percentage, and number of training shifts in the mother-baby unit at the time of pre- and post-optimization note completion.

**Pre-optimization training for first year residents (*n* = 5, 25%)**	**Pre-optimization training for second year residents (*n* = 5, 25%)**	**Post-optimization training for now second year residents (*n* = 5, 25%)**	**Post- optimization training for now third year residents (*n* = 5, 25%)**
6 weeks day shifts	6 weeks day shifts	6 weeks day shifts	6 weeks day shifts
2 weeks night shifts	4 weeks night shifts	4 weeks night shifts	4 weeks night shifts

### Intervention

The planned project was approved as Quality Improvement Pilot Project, and it was registered in the Quality Improvement Project Registry (QIPR) of our institution.

**Figure 3:**
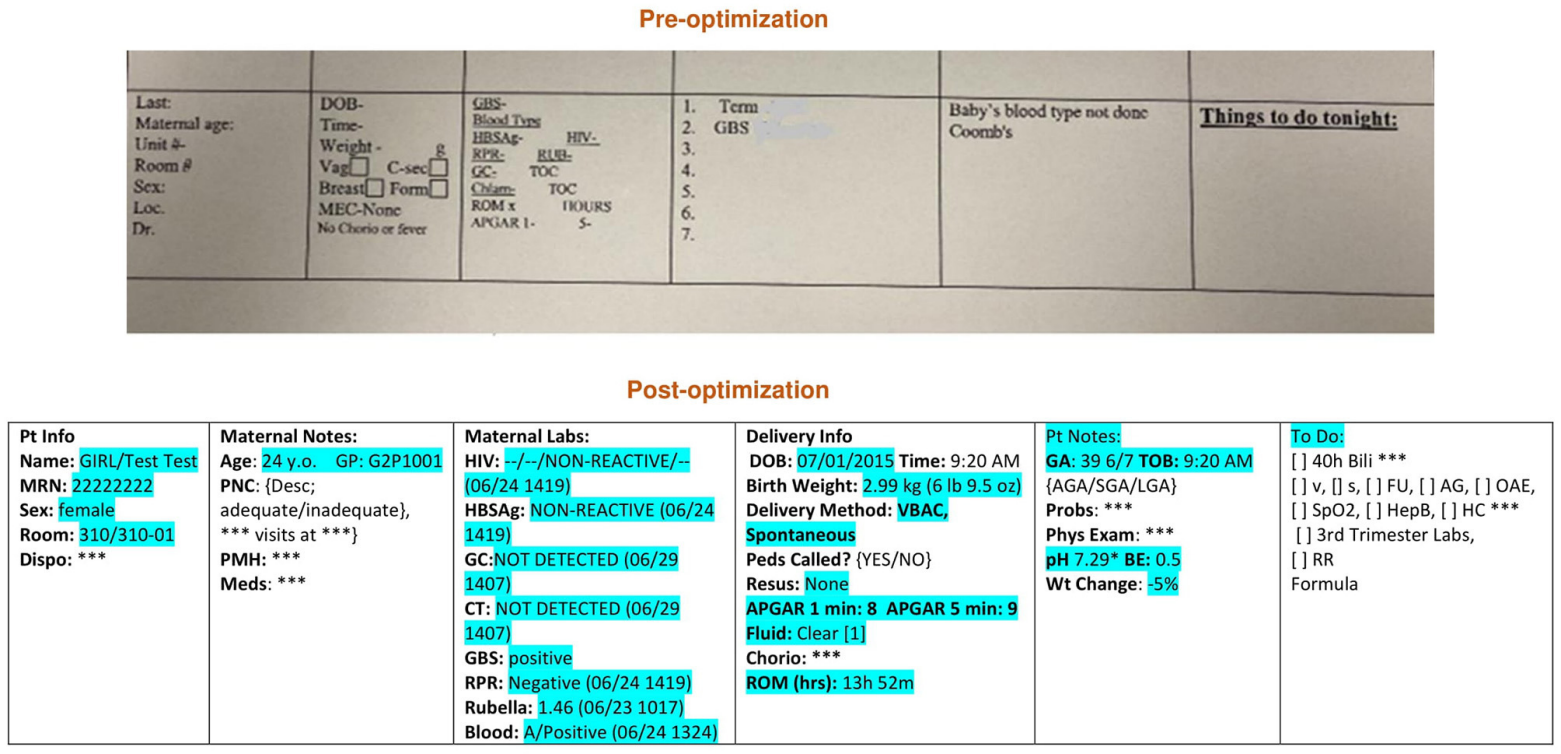
Hand-off list pre and post optimization. Loc, Location; DOB, Date of birth; Vag, Vaginal; C-sec, Cesarean section; Mec, Meconium; HIV, Human immunodeficiency virus; HBSAg, Hepatitis B antigen; GC, Gonorrhea; Chlam, Chlamydia; GBS, Group B streptococcus; RPR, Rapid plasma reagin; RUB, Rubella; Chorio, Chorioamnionitis; ROM, Rupture of membranes Blue highlight, Auto generated data; ***, Manual entry of data required; {}, Pick list; Epic codes are omitted; Y.o., Year old; Pt, Patient; Info, Information; Dispo, Disposition; PNC, Prenatal care; PMH, Past medical history; Meds, Medications; CT, Chlamydia; GBS, Group B streptococcus; RPR, Rapid plasma reagin; DOB, Date of birth; VBAC, Vaginal birth after cesarean section; Peds, Pediatrics, Resus, Resuscitation; GA, Gestational age; TOB, Time of birth; AGA, Appropriate for gestational age; SGA, Small for gestational age; LGA, Large for gestational age; Probs, Problems; Phys, Physical; BE, Base excess; Wt, Weight; Bili, bilirubin; V, Void; S, Stool; FU, Follow up; OAE, Otoacoustic emissions; SpO2, Oxygen saturation; Hep B, Hepatitis B vaccine; HC, Head circumference; RR, Red reflex.

The first step in the note optimization process was to identify all free text elements documented elsewhere in the medical records (e.g., delivery information, maternal data, and lab results) (Figures 1, 2). These elements were then replaced with dynamic links that automatically populate portions of the note. The second step was to identify the remaining free text elements that could be replaced with discrete data from pick lists. Pick lists are clickable drop-down lists that allow users to choose from lists of appropriate data points rather than manually typing them out. As part of this project, a brand-new hand-off list template was built in the EMR specifically for our MBU providers for patient sign-outs. The old hand-off list (HOL) was a simple database template that was not linked with EMR, and it required filling of almost all information on an infant and his/her mother manually (Figure 3). The new note templates were given a new and easily remembered name to increase ease of use and accessibility. The providers who care for infants admitted to the MBU were trained on how to find the new note templates. Also, template utilization tips were emailed to them, along with flyers placed in the MBU provider computer desk stations. The outcome of the project was the optimization of four physician note types in October 2015. These notes were history and physical (H and P), progress note (PN), discharge summary (DCS), and HOL. Only one template of each note type was to be used by all providers in the MBU.

### Measures

**Figure 4:**
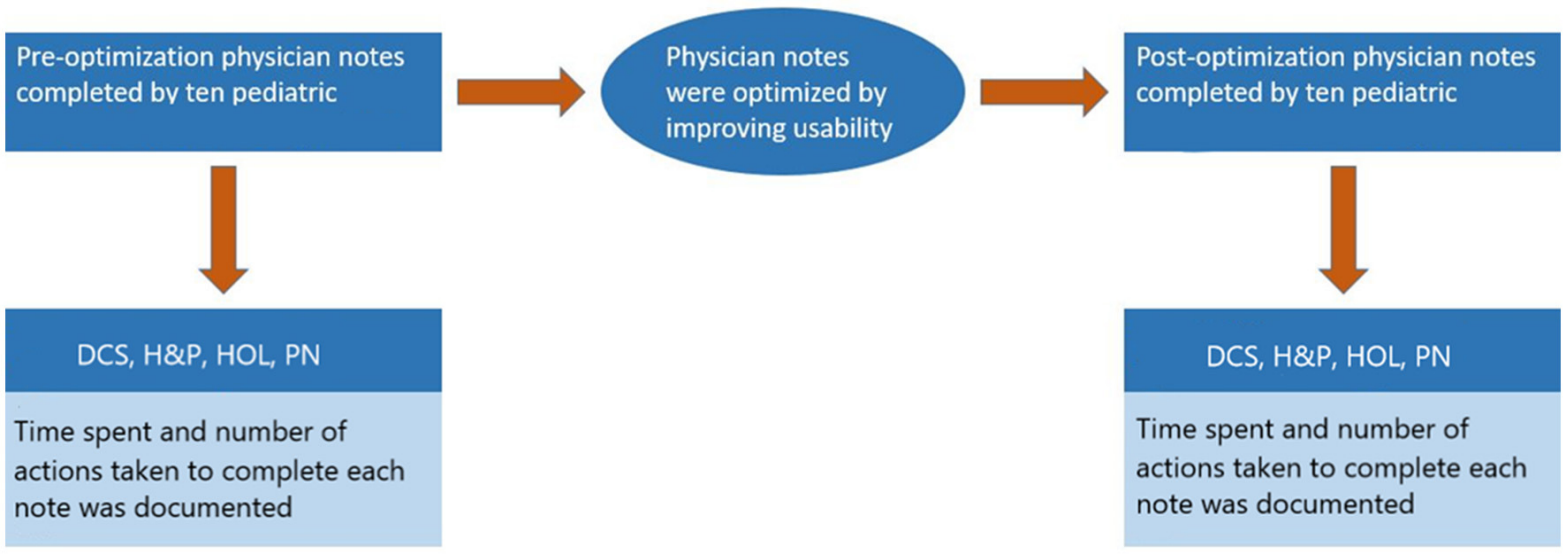
Flowchart of data collection methods.

Prior to optimization, during the second half of 2014, ten pediatric residents evenly divided in their first and second years of training (Table 1) were randomly chosen by the project's stakeholders to complete the four note types for one control newborn encounter (Figure 4). Each of the ten residents completed one note of each: H and P, PN, DCS, and HOL (scenarios of the assignments are included in Supplementary Material). The residents were then asked to collect and document discrete values. Next, they were given a signal to begin each note. This signal represented the start of a timer, which was stopped after they completed each note. Note completion time was tracked in seconds. The number of actions taken required to complete each note was counted. Actions taken were defined as: clicks, keystrokes, transitions, and mouse-keyboard switches.

**Figure 5:**
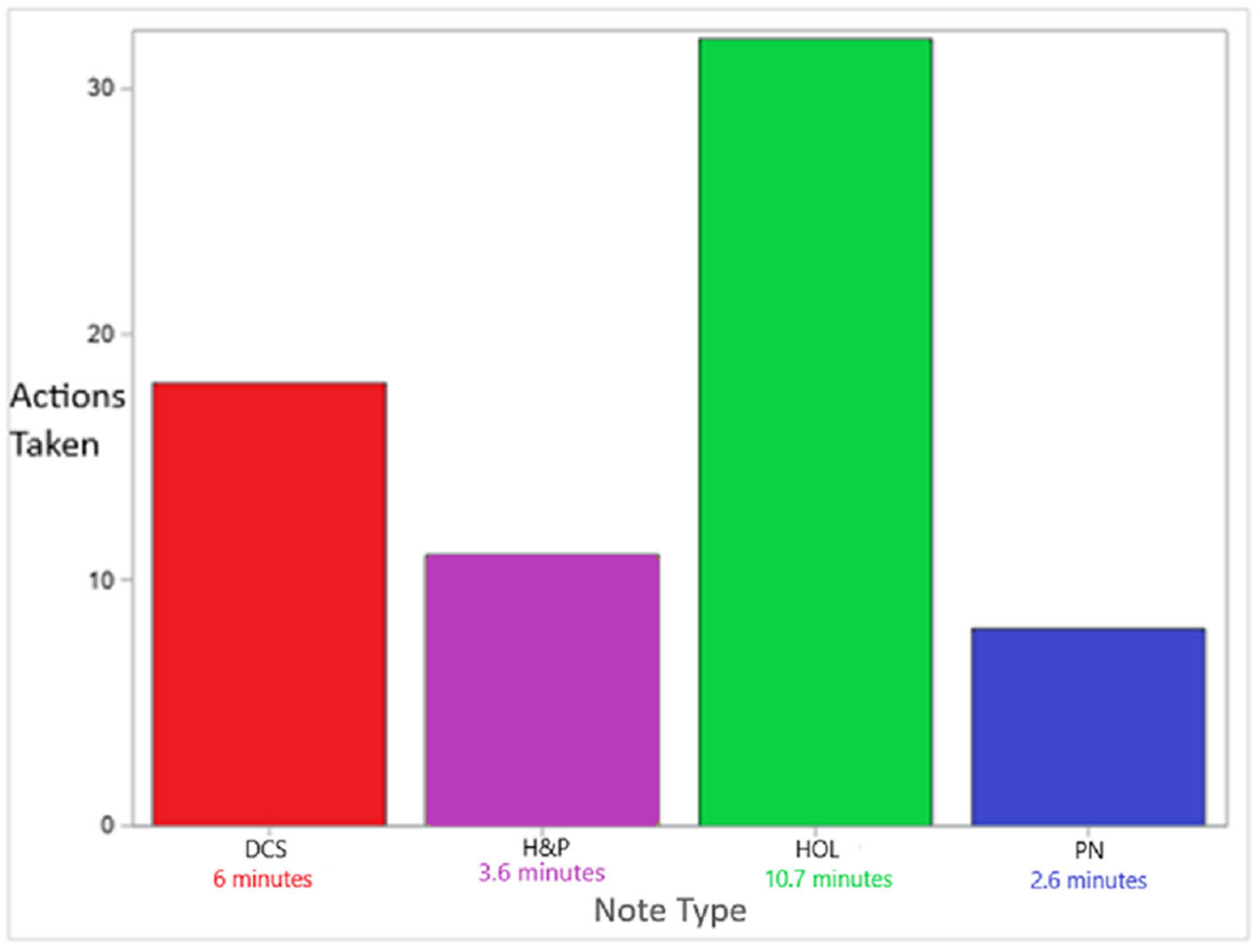
Saved number of actions taken and time by minute per note type Significant pre vs. post difference *p* = 0.002 per Wilcoxon Signed Rank test for each note type.

Post optimization, from October to December 2015, the same ten residents who completed the notes prior to optimization were again asked to complete notes, but this time with the newly optimized note templates (Figure 4). Again, each of the ten residents completed one of each note: H and P, PN, DCS, and HOL for the same control newborn encounter, which was used prior to optimization. Tracking the time and counting the actions taken were performed and recorded in the same manner as it had been previously collected (Figure 5).

None of the ten pediatric residents who completed the timed pre- and post-optimization notes was a stakeholder for this project.

### Analysis

The average length of stay (LOS) in the MBU is 2 days. During the entire LOS, every infant requires a minimum of three notes in total: H and P, PN, and DCS and daily updated HOL. However, to make the calculation simpler regarding HOL, we opted to record the time and count the actions taken required to complete the HOL only on the first day when the infant was born and admitted.

An anonymous survey form with three “yes/no” questions was sent to the MBU providers, which included pediatric residents, nurse practitioners, and pediatric faculty members, regarding accuracy, efficiency, and overall satisfaction of the new notes in comparison to the old notes. The survey link was sent to 46 providers' work emails, and they were given a week to complete it. The survey questions were:

1. Is the information generated from the new note templates more accurate than the information generated from the old note templates?
2. Were the new notes easier to complete than the old notes?
3. Are you, overall, satisfied with the new note templates as compared to the old templates?

To assess the efficiency of the new note templates and to check how much provider time was saved, we calculated the number of residents' minutes saved per month based on the average number of infants born in a given month. In order to do so, a report of the number of infants admitted to the MBU for the period of January 2016 to September 2019 (45 months) was requested from our data solution center. The generated report indicated that 9373 infants had been admitted to the MBU during the requested period.

## Results

Together, the actions taken saved when completing the new notes in comparison with the old notes were defined as follows: H and P: 11, PN: 8, DCS: 18, and HOL: 32. These numbers were exactly similar among all the ten residents (Figure 5). An exact Wilcoxon signed rank test was performed to compare pre- and post-optimization. A *p*-value of 0.002 was obtained for each measure of the notes, indicating statistically significant pre-post optimization comparison. The Bonferroni adjustment for multiple testing also showed statistical significance.

Time spent to complete the pre-optimization and post-optimization notes for the ten residents was recorded in s and converted to min (Table 2).

**Table 2:** Pre-optimization and post-optimization note-completion time in min by the ten pediatric residents.

**Residents & average**	**History and physical**		**Progress note**		**Discharge summary**		**Hand-off list**	
	**Pre**	**Post**	**Pre**	**Post**	**Pre**	**Post**	**Pre**	**Post**
**Resident 1**	14.5	11.2	11.2	8.4	16.8	11.5	16.4	6.1
**Resident 2**	15	10.8	10.8	8.2	17.3	10.9	15.5	4.7
**Resident 3**	14.6	10.7	10.7	8.4	17.4	10.5	16.2	5.2
**Resident 4**	14.8	11	11	8.7	16.8	10.8	17	6
**Resident 5**	14.2	10.9	10.9	9	17.2	11	16.6	5.7
**Resident 6**	14	10.7	10.7	8	16.2	11.2	15.4	5.2
**Resident 7**	14.8	10.7	10.7	8.1	16.6	10.8	16.2	4.9
**Resident 8**	14.5	11	11	8.5	17	10.7	15.4	5.4
**Resident 9**	14.1	11.1	11.1	8.1	17.1	11.3	15.9	5.5
**Resident 10**	14.7	10.9	10.9	8	16.9	10.6	16.3	5.3
**Average**	14.5	10.9	10.9	8.3	16.9	10.9	16.1	5.4

The number of residents completed the pre and post-optimization notes and the amount of training in MBU is seen in Table 1.

Completing the four new note templates took an average of 1,386 s less time when compared to completing the old ones for one infant. This was converted to min and it indicated that 23 min per infant was saved during the entire LOS, assuming, as mentioned earlier, that each infant required four notes, including an HOL, during the average two days LOS (Figure 5).

From January 2016 to September 2019 (45 months), there were 9,373 infants born in, averaging 208 infants per month. Reflecting the 23 min time saved per infant on the average 208 infants born per month during the study period, we estimated that 4,791 min were saved per month. Therefore, 80 h were saved per month, which is equal to 2.6 h saved per day.

Twenty-eight MBU providers completed the satisfaction survey, averaging 61% completion. Those who completed the survey showed an overwhelming 100% satisfaction with the new note templates (Table 3).

**Table 3:** Provider survey results.

**Questions**	**Yes % (*n*)**	**No% (*n*)**	**N/A% (*n*)**
Question 1: Is the new information generated in the new note templates more accurate than the information generated in the old note templates?	93% (26)	0% (0)	7% (2)
Question 2: Were the new notes easier to complete than the old notes?	100% (28)	0% (0)	0% (0)
Question 3: Are you overall satisfied with the new note templates as compared to the old templates?	100% (28)	0% (0)	0% (0)

## Discussion

### Summary

This study aimed to improve the efficiency and quality of documentation, and provider satisfaction simply by reforming the note templates in our MBU.

Optimizing the physician note templates to automatically extract relevant data and using pick lists reduced by 69 the number of clicks required to care for an infant in the MBU, which resulted in an estimated average of 4,791 min saved per month. This was achieved by simple improvement and optimization of the note templates. The providers were also substantially satisfied with the new templates.

### Interpretation

A hospital system in Saudi Arabia conducted a study on their EMR and found that it offered substantial benefits to healthcare professionals, as it improved access to information and increased healthcare professional productivity, efficiency, quality, and accuracy (11). In Australia, physician surveys concluded that improving healthcare provider recording behavior is important to improve the reliability and quality of electronically exchanged patient data (12). A study conducted in Canada on Canadian emergency departments found that if modern EMR is done correctly, it improves efficiency, communication, and patient safety. However, poor EMR implementation will cause provider burnout and inefficiency (13).

Based on previous examples, we can confidently state that EMR is an important tool in healthcare around the world. It should ideally improve patient care, communication, provider efficiency, and productivity. The goal and benefits of EMRs are the same worldwide and across all departments in both inpatient and ambulatory settings. In 2010, the University of Washington in Seattle found that the main barrier in transitioning patient medical records from paper to electronic physician inpatient notes was the time required to enter the notes that contained data-rich templates (14). The same study addressed the importance of standardizing documentation using templates to improve provider efficiency. An article published in 2018 involved a survey of eighty-four orthopedic residents to assess their perception of the purpose of EMR. It concluded that although the residents understand EMR values on patient care and safety, some felt that they could not utilize EMR efficiently because of time constraints (15).

Our project successfully made simple adjustments to the existing EMR and improved physician note usability and documentation. It also succeeded in estimating the amount of hours saved on a daily basis. The significance of this accomplishment in the healthcare system, specifically in an academic setting, is resident involvement, their patient care, and education while also complying with their duty hours (16). In 2016, Ham et al. was able to increase direct patient care time and decrease rates of duty hour violations among his surgery residents simply by improving their round report usability (16).

The literature is clear that EMR implementation is necessary to improve healthcare in general, as stated above. However, not enough literature on how we can improve the usability of EMR and optimize it to reach high levels of patient safety and provider satisfaction exists. Michael Kavuma defined usability as the effectiveness, efficiency, and satisfaction with which specific users can achieve a specific set of tasks in a particular environment (17). Maximizing the use of templates and smart phrases is the second of ten EMR strategies for efficient documentation published by Jay Winer (18). Pierce et al. optimized their EMR documentation by exploring the best practices, which resulted in them seeing one more patient per day in their ambulatory care setting (19).

In this project, we worked on the usability of our EMR, optimized the physician notes, and improved the workflow in the MBU. We were able to save time on a daily basis, enabling us to spend more time in direct patient care. We also demonstrated an optimal and efficient EMR documentation and improvement in provider satisfaction.

### Limitations

Our study limitations: (1) the resident sample size of ten was small and arbitrarily chosen; (2) there is a slight possibility of bias in time measurements due to repetition; (3) 25% of the residents who completed the pre-optimization notes had slightly less training in the MBU than the rest of the residents; (4) the MBU provider survey was limited to three “yes/no,” leading questions with no option for comments by providers; (5) this project was conducted in a single center with standardized MBU workflow; therefore, there is a possibility that our approach might not apply to other units and elsewhere in the EMR.

## Conclusions

This study concluded that optimizing physician note templates saved an average of 2.6 h per day and improved provider satisfaction.

Workflow in MBUs can be made more efficient by integrating obstetric and pediatric charts with elements of standardized data to reduce duplication of documentation. A similar approach may help in other multidisciplinary care settings. More projects on EMR usability should be taken across healthcare to serve as positive tools for patient care and provider satisfaction.

## Data Availability Statement

The original contributions presented in the study are included in the article/Supplementary Material, further inquiries can be directed to the corresponding author/s.

## Ethics Statement

The studies involving human participants were reviewed and approved by University of Florida. Written informed consent from the participants' legal guardian/next of kin was not required to participate in this study in accordance with the national legislation and the institutional requirements.

## Author Contributions

RA is a stakeholder and attended the meetings with EMR analytics, formulated the hypothesis, structured the study design, registered it in QIPR and the data solution center, supervised note implementation and production process, organized and conducted the time tables, Supplementary Material, interpreted the results, and drafted the article. JH edited the original manuscript, made revisions, and contributed in discussions. KW supervised the manuscript process and edited the article.

## Conflict of Interest

The authors declare that the research was conducted in the absence of any commercial or financial relationships that could be construed as a potential conflict of interest.

## Publisher's Note

All claims expressed in this article are solely those of the authors and do not necessarily represent those of their affiliated organizations, or those of the publisher, the editors and the reviewers. Any product that may be evaluated in this article, or claim that may be made by its manufacturer, is not guaranteed or endorsed by the publisher.
