# Supplementary material for: Saving Time for Patient Care by Optimizing Physician Note Templates: A Pilot Study
Source: Front Digit Health. 2022 Jan 13;3:772356. doi: 10.3389/fdgth.2021.772356 (PMC8792616; doi:10.3389/fdgth.2021.772356)
Supplement: Supplementary file 1 [file Data_Sheet_1.PDF]

Department of Pediatrics
Newborn Nursery
Admission Note

Admit Date: 07/01/2015 9:20 AM

Subjective

Date of birth: 07/01/2015
Time of birth: 0920
Admit date: 07/01/2015

GA Dates: Gestational Age: 39w6d
Apgar scores:
APGAR 1 min: 9
APGAR 5 min: 9

Maternal Perinatal History:

Mother's name: Test, Test
Mother's DOB: 4/7/1997
Mother's Age: 24 y.o.
GP status: G2P2002

Prenatal labs:
HIV:
Information for the patient's mother: *Test, Test [11111111]*
{POSITIVE/NEGATIVE/UNKNOWN}

HepBSAg:
Information for the patient's mother: *Test, Test [11111111]*
{POSITIVE/NEGATIVE/UNKNOWN}

GC:
Information for the patient's mother: *Test, Test [11111111]*
{POSITIVE/NEGATIVE/UNKNOWN}

CT:
Information for the patient's mother: *Test, Test [11111111]*
{POSITIVE/NEGATIVE/UNKNOWN}

GBS: {Positive/Negative/Pending/Never Tested}

******Please make sure to verify ALL maternal prenatal lab results before signing discharge summary. Delete this line once completed.***

RPR:
Information for the patient's mother: *Test, Test [11111111]*
{POSITIVE/NEGATIVE/UNKNOWN}

Rubella:
Information for the patient's mother: *Test, Test [11111111]*
{POSITIVE/NEGATIVE/UNKNOWN}

Blood:
Information for the patient's mother: *Test, Test 11111111]*
Results in Past 300 Days

Result Component	Current Result	Ref Range	Previous Result	Ref Range
ABO Grouping	A (07/01/2015)		A (07/01/2014)	
Rh Type	Positive (07/01/2015)		Positive (07/01/2014)	

Prenatal care: {Desc; adequate/inadequate}, *** visits at ***}

Pregnancy complications: ***

Labor Events

Preterm labor?: No
GBS Status: positive
Antibiotics for GBS given?: Yes
Rupture date: 07/01/2015 Rupture time: 09:00
Rupture type: Artificial
Induction: AROM, Oxytocin
Augmentation: None

Additional complications:	OB: DELIVERY - COMPLICATIONS
	History of cesarean section

Perinatal complications: ***

Prenatal Medications: ***

Delivery Method: VBAC, Spontaneous

ROM: ***

Peds called: {yes no}

Resuscitation: ***

Cord Blood pH: ***

Objective

Birth Weight: 2990 g (6 lb 9.5 oz) (Filed from Delivery Summary), {AGA/SGA/LGA}
Length:
Ht Readings from Last 1 Encounters:
07/01/2015 48.9 cm (19.25") (27 %, Z= -0.62)*

* Growth percentiles are based on Fenton (Girls, 22-50 Weeks) data.
27 %ile (Z= -0.62) based on Fenton (Girls, 22-50 Weeks) Length-for-age data based on Length recorded on 07/01/2015.
Head Circumference:

HC Readings from Last 1 Encounters:
07/01/2015 31.5 cm (12.4") (1 %, Z= -2.26)*

* Growth percentiles are based on Fenton (Girls, 22-50 Weeks) data.
1 %ile (Z= -2.26) based on Fenton (Girls, 22-50 Weeks) head circumference-for-age based on Head Circumference recorded on 07/01/2015.

Admission Exam date and time: 07/01/2015 10:00 AM

General: alert, in no acute distress, no dysmorphic features
Head: fontanelles open, soft, flat and normal size
Eyes: sclera white; pupils equal and reactive; **red reflex present both eyes**
Ears: well-positioned, well-formed pinnae, no preauricular sinuses or tags
Nose: clear, normal mucosa
Mouth: normal tongue, palate intact
Neck: normal structure
Chest: lungs clear to auscultation, unlabored breathing
Heart: regular rate and rhythm; no murmurs
Abdomen/Anus: soft, non-tender, non-distended; without masses or hepatosplenomegaly; anus patent; umbilical stump clean and dry
Pulses: strong equal femoral pulses, brisk capillary refill
Hips: negative Barlow, Ortolani, gluteal creases equal
GU: {male female} ***please list penis length in cm. if female, delete this phrase***
Extremities: well-perfused, warm and dry; clavicles intact
Spine: normal, symmetric, no sacral tufts, tags or dimples
Skin: warm, dry and intact
Neurologic: easily aroused; good symmetric tone and strength; positive root and suck; symmetric normal reflexes

Patient Active Problem List
Diagnosis
- Single liveborn infant delivered vaginally

Assessment

Baby GIRL/Test, Test is an Gestational Age: 39w6d week {AGA/SGA/LGA} female infant born via {DELIVERY METHOD}, now 1 hour.

Plan

Continue neonatal care.

- Watch for infant to void and stool
- Hepatitis B immunization
- Erythromycin ointment to eyes
- Vitamin K injection
- Hearing test (OAE)
- Congenital heart disease screen (upper/lower extremity SpO2 check @ 24 hrs)

- 40 hr bili and newborn metabolic screen *** @ ***
- Infant circumcision {is/is not} appropriate given physiologic status, anatomy and length of phallus. *****Delete this bullet point for females**

Will provide anticipatory guidance.

Will need follow-up with PCP 1-2 days after discharge.

Me, MD
07/01/2015 10:30 AM

Example 1: History and physical pre-optimization

Blue highlight: Auto generated data. ***: Manual entry of data required. { }: Pick list. Epic codes are omitted.

Yo: Year old. GP: Gravida para. HIV: Human immunodeficiency virus, HBSAg: Hepatitis B antigen. GC: Gonorrhea. CT: Chlamydia. GBS: Group B streptococcus. RPR: Rapid plasma reagin. VBAC: Vaginal birth after cesarean section. ROM: Rupture of membranes. Peds: Pediatrics. GA: Gestational age. AGA: Appropriate for gestational age. SGA: Small for gestational age. LGA: Large for gestational age. OAE: Otoacoustic emissions. SpO2: Oxygen saturation. Bili: Bilirubin.
