# Supplementary material for: Saving Time for Patient Care by Optimizing Physician Note Templates: A Pilot Study
Source: Front Digit Health. 2022 Jan 13;3:772356. doi: 10.3389/fdgth.2021.772356 (PMC8792616; doi:10.3389/fdgth.2021.772356)
Supplement: Supplementary file 3 [file Data_Sheet_3.PDF]

**Department of Pediatrics**
**Newborn Nursery**
**Progress Note**

## Subjective

**Diet:** {breast, formula, breast and formula, ***}
**Intake/Output:**
Overnight, Baby girl Test Test did ***. No other acute issues at this time.

| Date | 07/01/15 0700 - 07/02/15 0659 | | | | 07/02/21 0700 - 07/02/21 0659 | | | |
|---|---|---|---|---|---|---|---|---|
| Shift | 0700-1459 | 1500-2259 | 2300-0659 | 24 Hour Total | 0700-1459 | 1500-2259 | 2300-0659 | 24 Hour Total |
| INTAKE | | | | | | | | |
| P.O. | | 74 | 25 | 99 | | | | |
| Formula - P.O. (mL) | | 74 | 25 | 99 | | | | |
| Shift Total | | 74 | 25 | 99 | | | | |
| OUTPUT | | | | | | | | |
| Urine | | | | | | | | |
| Urine Occurrence | | | 1 x | 1 x | | | | |
| Stool | | | | | | | | |
| Stool Occurrence | | | 1 x | 1 x | | | | |
| Shift Total | | | | | | | | |
| NET | | 74 | 25 | 99 | | | | |

## Objective

| | 07/01/15 2130 | 07/02/15 0330 | 07/02/15 0800 |
|---|---|---|---|
| BP: | | | |
| Pulse: | 128 | 120 | 160 |
| Resp: | 52 | 40 | 42 |
| Temp: | 37.2 °C (99 °F) | 37.1 °C (98.8 °F) | 37.4 °C (99.3 °F) |
| TempSrc: | Axillary | Axillary | Axillary |
| Weight: | | | |
| Height: | | | |
| HC: | | | |

**Weight:** ***, **Weight change since birth:** ***

**Physical Exam:**
**General:** alert, in no acute distress, no dysmorphic features
**Head:** fontanelles open, soft, flat and normal size
**Eyes:** sclerae white, no discharge or injection

**Ears:** well-positioned, well-formed pinnae
**Nose:** clear, normal mucosa
**Mouth:** normal tongue, palate intact
**Neck:** normal structure
**Chest:** lungs clear to auscultation, unlabored breathing
**Heart:** regular rate and rhythm; no murmurs
**Abdomen/Anus:** soft, non-tender, non-distended; no HSM or masses, umbilical stump clean and dry
**Pulses:** strong equal femoral pulses, brisk capillary refill
**Hips:** negative Barlow & Ortolani, gluteal creases equal
**GU:** normal *** genitalia
**Extremities:** well-perfused, warm and dry
**Spine:** normal, symmetric
**Skin:** warm, dry and intact
**Neurologic:** easily aroused; good symmetric tone and strength; positive root and suck; symmetric normal reflexes, no focal defects

**Labs:**
No results found for this visit on 07/02/2015 (from the past 24 hour(s)).

**Patient Active Problem List**

| Diagnosis |
|---|
| • Single liveborn infant delivered vaginally |

## Assessment

Baby girl Test Test is a 40w 2d {AGA/SGA/LGA} female infant, born via Delivery Method: VBAC, Spontaneous on 07/01/15, now 1 days.

## Plan

- Baby has voided and stooled.
- Hearing test (OAE)
- Congenital heart screen - SpO2 check @ 24 hrs: ***
- 40hr bili *** @ ***

**Immunization History**

| Administered | Date(s) Administered |
|---|---|
| • Hepatitis B Vaccine (Peds/Adol 3-dose), IM | 07/01/2015 |

**Anticipatory guidance provided on:** ***

**Will follow up with *** 1-2 days after discharge.**

Me, MD
07/02/2015 10:30 AM

**Example 3: Progress note pre-optimization**

Blue highlight: Auto generated data. ***: Manual entry of data required. { }: Pick list. Epic codes are omitted.

VBAC: Vaginal birth after cesarean section. AGA: Appropriate for gestational age. SGA: Small for gestational age. LGA: Large for gestational age. OAE: Otoacoustic emissions. SpO2: Oxygen saturation. Bili: Bilirubin.
