# Supplementary material for: Saving Time for Patient Care by Optimizing Physician Note Templates: A Pilot Study
Source: Front Digit Health. 2022 Jan 13;3:772356. doi: 10.3389/fdgth.2021.772356 (PMC8792616; doi:10.3389/fdgth.2021.772356)
Supplement: Supplementary file 4 [file Data_Sheet_4.PDF]

Department of Pediatrics
Newborn Nursery
Progress Note

Subjective

Diet: {breast, formula, breast and formula, ***}
Intake/Output:
Overnight, Baby girl Test Test did ***. No other acute issues at this time.

Date	07/01/15 0700 - 07/02/15 0659				07/02/15 0700 – 07/012/15 0659			
Shift	0700-1459	1500-2259	2300-0659	24 Hour Total	0700-1459	1500-2259	2300-0659	24 Hour Total
INTAKE								
P.O.		74	25	99				
Formula - P.O. (mL)		74	25	99				
Shift Total		74	25	99				
OUTPUT								
Urine								
Urine Occurrence			1 x	1 x				
Stool								
Stool Occurrence			1 x	1 x				
Shift Total								
NET		74	25	99				

Objective

Vitals:

	07/01/15 2130	07/02/15 0125	07/02/15 0330	07/02/15 0800
BP:				
Pulse:	128		120	160
Resp:	52		40	42
Temp:	37.2 °C (99 °F)		37.1 °C (98.8 °F)	37.4 °C (99.3 °F)
TempSrc:	Axillary		Axillary	Axillary
Weight:		2880 g (6 lb 5.6 oz)		
Height:				
HC:				

Weight: 2880 g (6 lb 5.6 oz) (07/02 0125), **Weight change since birth:** -4%

Physical Exam:
General: alert, in no acute distress, no dysmorphic features
Head: fontanelles open, soft, flat and normal size
Eyes: sclerae white, no discharge or injection

Ears: well-positioned, well-formed pinnae
Nose: clear, normal mucosa
Mouth: normal tongue, palate intact
Neck: normal structure
Chest: lungs clear to auscultation, unlabored breathing
Heart: regular rate and rhythm; no murmurs
Abdomen/Anus: soft, non-tender, non-distended; no HSM or masses, umbilical stump clean and dry
Pulses: strong equal femoral pulses, brisk capillary refill
Hips: negative Barlow & Ortolani, gluteal creases equal
GU: normal *** genitalia
Extremities: well-perfused, warm and dry
Spine: normal, symmetric
Skin: warm, dry and intact
Neurologic: easily aroused; good symmetric tone and strength; positive root and suck; symmetric normal reflexes, no focal defects

Labs:
No results found for this visit on 07/02/2015 (from the past 24 hour(s)).

Patient Active Problem List

Diagnosis
• Single liveborn infant delivered vaginally

Assessment

Baby girl Test Test is a 40w 2d {AGA/SGA/LGA} female infant, born via Delivery Method: VBAC, Spontaneous on 07/01/15, now 1 days.

Plan

- Baby has voided and stooled.
- Hearing test (OAE)
- Baby's 24 hour CCHD Screen: SpO2: Pre-Ductal (Right Hand): 97 % and SpO2: Post-Ductal (Either Foot): 98 % Critical Congenital Heart Defect Score: Negative
- 40hr bili *** @ ***

Immunization History

Administered	Date(s) Administered
• Hepatitis B Vaccine (Peds/Adol 3-dose), IM	07/01/2015

Anticipatory guidance provided on: risks of co-sleeping, **breast feeding*****, formula feeding, bowel movements, bathing and cord care, **circumcision care*****, dental care, temperature, activity, smoking, stimulation, back to sleep, shaken baby syndrome, car

seat safety, baby blues and when to call the doctor. Standard newborn care instructions and anticipatory guidance were provided to the mother prior to discharge.

Will follow up with * 1-2 days after discharge.**

Me, MD
07/02/2015 11:00 AM

Example 4: Progress note post-optimization

Blue highlight: Auto generated data. ***: Manual entry of data required. { }: Pick list. Epic codes are omitted.

VBAC: Vaginal birth after cesarean section. AGA: Appropriate for gestational age. SGA: Small for gestational age. LGA: Large for gestational age. OAE: Otoacoustic emissions. SpO2: Oxygen saturation. Bili: Bilirubin.
