# Supplementary material for: Saving Time for Patient Care by Optimizing Physician Note Templates: A Pilot Study
Source: Front Digit Health. 2022 Jan 13;3:772356. doi: 10.3389/fdgth.2021.772356 (PMC8792616; doi:10.3389/fdgth.2021.772356)
Supplement: Supplementary file 5 [file Data_Sheet_5.PDF]

Department of Pediatrics
Newborn Nursery
Discharge Summary

Date of birth: 07/01/2015
Time of birth: 0920
Admit date: 07/01/2015

Discharge date and time: 07/03/2015 10:30 AM

GA Dates: Gestational Age: 39w6d
Apgar scores:
APGAR 1 min: 9
APGAR 5 min: 9

Maternal Perinatal History:

Mother's name: Test, Test
Mother's DOB: 4/7/1997
Mother's Age: 24 y.o.
GP status: G2P2002

Prenatal labs:

HIV: {POSITIVE/NEGATIVE/UNKNOWN}

HepBSAg: {POSITIVE/NEGATIVE/UNKNOWN}

GC: {POSITIVE/NEGATIVE/UNKNOWN}

CT: {POSITIVE/NEGATIVE/UNKNOWN}

GBS: {Positive/Negative/Pending/Never Tested} ***___Please make sure to verify ALL maternal prenatal lab results before signing discharge summary. Delete this line once completed.___***

RPR: {reactive/non-reactive}

Rubella: {Desc; immune/not/unknown}

Blood: {misc;blood types}

Prenatal care: {Desc; adequate/inadequate}, *** visits at ***

Pregnancy complications: ***

Perinatal complications: ***

Prenatal medications: ***

Delivery Method: VBAC, Spontaneous

ROM: ***

Peds called: {JX YES/NO}

Resuscitation: ***

Admission Physical:
Birth weight: 2990 g (6 lb 9.5 oz) (Filed from Delivery Summary)
{AGA/SGA/LGA}

Birth Weight: 2990 g (6 lb 9.5 oz) (Filed from Delivery Summary), {AGA/SGA/LGA}
Length:
Ht Readings from Last 1 Encounters:
07/01/2015 48.9 cm (19.25") (27 %, Z= -0.62)*

* Growth percentiles are based on Fenton (Girls, 22-50 Weeks) data.
27 %ile (Z= -0.62) based on Fenton (Girls, 22-50 Weeks) Length-for-age data based on Length recorded on 07/01/2015.
Head Circumference:
HC Readings from Last 1 Encounters:
07/01/2015 31.5 cm (12.4") (1 %, Z= -2.26)*

* Growth percentiles are based on Fenton (Girls, 22-50 Weeks) data.
1 %ile (Z= -2.26) based on Fenton (Girls, 22-50 Weeks) head circumference-for-age based on Head Circumference recorded on 07/01/2015.

Admission physical exam was normal except for ***. Red reflex was present bilaterally on admission.

Laboratory/Diagnostic Tests:

Results for orders placed or performed during the hospital encounter of 10/26/21 (from the past 72 hour(s))
Blood Gas Arterial, Cord
Collection Time: 07/01/2015 10:20 AM

Result	Value
Specimen Type	Other
Site	V.CORD
Allens Test	No
pH, Cord Art	7.35
pO2, Cord Art	41.0
Calculated Bicarbonate	24.8
Calculated Base Excess	-1.1 (L)
Calculated O2	73.2
PCO Cord Arterial	45

Blood Gas Arterial, Cord
Collection Time: 07/01/2015 10:20 AM

Result	Value
Specimen Type	Other
Site	A.CORD
Allens Test	No
pH, Cord Art	7.29 (L)
pO2, Cord Art	19.0
Calculated Bicarbonate	28.4
Calculated Base Excess	0.5
Calculated O2	23.0
PCO Cord Arterial	59

POCT bilirubinometry
Collection Time: 07/03/2015 1:21 AM

Result	Value
Bilirubin Tc	7.6

Nutrition: {breast, formula, breast and formula, ***}

Discharge Physical:

Vitals:

	09/02/2015 30	09/03/15 0125	09/03/15 0330	09/03/15 0800
BP:				
Pulse:	128		120	160
Resp:	52		40	42
Temp:	37.2 °C (99 °F)		37.1 °C (98.8 °F)	37.4 °C (99.3 °F)
TempSrc:	Axillary		Axillary	Axillary
Weight:		2830 g (6 lb 3.8 oz)		
Height:				
HC:				

Discharge weight: ***
Weight Change Since Birth: ***

General: alert, in no acute distress, no dysmorphic features
Head: fontanelles open, soft, flat and normal size, **HC *** cm**
Eyes: sclera white; pupils equal and reactive
Ears: well-positioned, well-formed pinnae, no preauricular sinuses or tags
Nose: clear, normal mucosa
Mouth: normal tongue, palate intact
Neck: normal structure
Chest: lungs clear to auscultation, unlabored breathing
Heart: regular rate and rhythm; no murmurs
Abdomen/Anus: soft, non-tender, non-distended; without masses or hepatosplenomegaly; anus patent; umbilical stump clean and dry
Pulses: strong equal femoral pulses, brisk capillary refill
Hips: negative Barlow, Ortolani, gluteal creases equal
GU: normal *** genitalia
Extremities: well-perfused, warm and dry; clavicles intact
Spine: normal, symmetric, no sacral tufts, tags or dimples
Skin: warm, dry and intact
Neurologic: easily aroused; good symmetric tone and strength; positive root and suck; symmetric normal reflexes

Newborn Hearing Screen (OAE):
****

Immunization History

Administered	Date(s) Administered
• Hepatitis B Vaccine (Peds/Adol 3-dose), IM	07/01/2015

Anticipatory guidance performed: ***

Patient Active Problem List

Diagnosis
• Single liveborn infant delivered vaginally

Hospital Course: Baby GIRL/Test Test was born on 07/01/2015 at 0920 at Gestational Age: 39w6d via Delivery Method: VBAC, Spontaneous to a 24 y.o. G2P2002 . Peds {WAS/WAS NOT} called and no resuscitation was required. *** Baby did well overall and has fed, voided and stooled appropriately. Mom received anticipatory guidance on caring for her new baby. No other acute issues arose during this admission. Baby deemed medically stable for discharge. Baby to f/u with *** in 1-2 days.

- Baby's 24 hour and ***% in both upper and lower extremities.
- Baby's 40 hour bilirubin was ***
- Birth weight was 2990 g (6 lb 9.5 oz) (Filed from Delivery Summary)
- Discharge weight was ***
- Admission weight change of ***
- Infant {WAS/WAS NOT:"was not"} circumcised during his newborn hospital stay. *****delete this bullet point for females*****

Studies pending: {None:"None"}
Special Follow-up Needed: {None:"None"}

Discharge Instructions: Standard newborn care instructions and anticipatory guidance were provided to the mother prior to discharge.

Me, MD
07/03/2015 10:30 AM

Example 5: Discharge summary pre-optimization

Blue highlight: Auto generated data. ***: Manual entry of data required. { }: Pick list. Epic codes are omitted.
Yo: Year old. GP: Gravida para. HIV: Human immunodeficiency virus, HBSAg: Hepatitis B antigen. GC: Gonorrhea. CT: Chlamydia. GBS: Group B streptococcus. RPR: Rapid plasma reagin. VBAC: Vaginal birth after cesarean section. ROM: Rupture of membranes. Peds: Pediatrics. GA: Gestational age. AGA: Appropriate for gestational age. SGA: Small for gestational age. LGA: Large for gestational age. OAE: Otoacoustic emissions. SpO2: Oxygen saturation. Bili: Bilirubin.
