# Supplementary material for: Saving Time for Patient Care by Optimizing Physician Note Templates: A Pilot Study
Source: Front Digit Health. 2022 Jan 13;3:772356. doi: 10.3389/fdgth.2021.772356 (PMC8792616; doi:10.3389/fdgth.2021.772356)
Supplement: Supplementary file 6 [file Data_Sheet_6.PDF]

Department of Pediatrics
Newborn Nursery
Discharge Summary

Admit Date: 07/01/2015 9:20 AM

Subjective

Date of birth: 07/01/2015
Time of birth: 0920
Admit date: 07/01/2015

GA Dates: Gestational Age: 39w6d
Apgar scores:
APGAR 1 min: 9
APGAR 5 min: 9

Maternal Perinatal History:

Name: Test,Test   Age: 24 y.o.   GP Status: G2P2002

HIV: --/--/NON-REACTIVE/-- (06/24 1419)
HBSAg: NON-REACTIVE (06/24 1419)
GC:NOT DETECTED (06/29 1407)
CT: NOT DETECTED (06/29 1407)
GBS: positive
RPR: Negative (06/24 1419)
Rubella: 1.46 (06/23 1017)
Blood: A/Positive (06/24 1324)

Prenatal care: {Desc; adequate/inadequate}, *** visits at ***}

Pregnancy complications: ***

Labor Events

Preterm labor?: No
GBS Status: positive
Antibiotics for GBS given?: Yes
Rupture date: 07/01/2015   Rupture time: 09:00
Rupture type: Artificial
Induction: AROM, Oxytocin
Augmentation: None

Additional complications:	OB: DELIVERY - COMPLICATIONS
	History of cesarean section

Perinatal complications: ***

Prenatal Medications:

Medications Prior to Admission

Medication	Sig
• [DISCONTINUED] aspirin 81 MG Oral Tablet Chewable	Chew 1 tablet daily.
• ferrous sulfate 325 (65 FE) MG Oral Tablet	Take 1 tablet by mouth daily (with breakfast).
• prenatal multivitamin (VITAFOL-OB) Oral Tablet	Take 1 tablet by mouth daily.

Delivery Method: VBAC, Spontaneous

ROM: 13h 52m

Peds called: {JX YES/NO}

Resuscitation: None

Cord Blood pH:

pH, Cord Art

Date	Value	Ref Range	Status
07/01/2015	7.29 (L)	7.32 - 7.42	Final

Calculated Base Excess

Date	Value	Ref Range	Status
07/01/2015	0.5	0 - 2 MMOL/L	Final

Admission Physical:

Birth weight: 2990 g (6 lb 9.5 oz) (Filed from Delivery Summary)
{AGA/SGA/LGA}

Birth Weight: 2990 g (6 lb 9.5 oz) (Filed from Delivery Summary), {AGA/SGA/LGA}
Length:
Ht Readings from Last 1 Encounters:
07/01/2015 48.9 cm (19.25") (27 %, Z= -0.62)*

* Growth percentiles are based on Fenton (Girls, 22-50 Weeks) data.
27 %ile (Z= -0.62) based on Fenton (Girls, 22-50 Weeks) Length-for-age data based on Length recorded on 07/01/2015.
Head Circumference:
HC Readings from Last 1 Encounters:
07/01/2015 31.5 cm (12.4") (1 %, Z= -2.26)*

* Growth percentiles are based on Fenton (Girls, 22-50 Weeks) data.
1 %ile (Z= -2.26) based on Fenton (Girls, 22-50 Weeks) head circumference-for-age based on Head Circumference recorded on 07/01/2015.

Admission physical exam was normal except for ***. Red reflex was present bilaterally on admission.

Laboratory/Diagnostic Tests:

Results for orders placed or performed during the hospital encounter of 10/26/21 (from the past 72 hour(s))
Blood Gas Arterial, Cord
Collection Time: 07/01/2015 10:20 AM

Result	Value
Specimen Type	Other

Site	V.CORD
Allens Test	No
pH, Cord Art	7.35
pO2, Cord Art	41.0
Calculated Bicarbonate	24.8
Calculated Base Excess	-1.1 (L)
Calculated O2	73.2
PCO Cord Arterial	45

Blood Gas Arterial, Cord

Collection Time: 07/01/2015 10:20 AM

Result	Value
Specimen Type	Other
Site	A.CORD
Allens Test	No
pH, Cord Art	7.29 (L)
pO2, Cord Art	19.0
Calculated Bicarbonate	28.4
Calculated Base Excess	0.5
Calculated O2	23.0
PCO Cord Arterial	59

POCT bilirubinometry

Collection Time: 07/03/2015 1:21 AM

Result	Value
Bilirubin Tc	7.6

Imaging: No results found.
Nutrition: {breast, formula, breast and formula, ***}

Discharge Physical:

Vitals:

	09/02/2015 30	09/03/15 0125	09/03/15 0330	09/03/15 0800
BP:				
Pulse:	128		120	160
Resp:	52		40	42
Temp:	37.2 °C (99 °F)		37.1 °C (98.8 °F)	37.4 °C (99.3 °F)
TempSrc:	Axillary		Axillary	Axillary
Weight:		2830 g (6 lb 3.8 oz)		
Height:				
HC:				

Discharge weight: ***
Weight Change Since Birth: ***

General: alert, in no acute distress, no dysmorphic features
Head: fontanelles open, soft, flat and normal size, **HC *** cm**
Eyes: sclera white; pupils equal and reactive
Ears: well-positioned, well-formed pinnae, no preauricular sinuses or tags
Nose: clear, normal mucosa
Mouth: normal tongue, palate intact

Neck: normal structure
Chest: lungs clear to auscultation, unlabored breathing
Heart: regular rate and rhythm; no murmurs
Abdomen/Anus: soft, non-tender, non-distended; without masses or hepatosplenomegaly; anus patent; umbilical stump clean and dry
Pulses: strong equal femoral pulses, brisk capillary refill
Hips: negative Barlow, Ortolani, gluteal creases equal
GU: normal female genitalia
Extremities: well-perfused, warm and dry; clavicles intact
Spine: normal, symmetric, no sacral tufts, tags or dimples
Skin: warm, dry and intact
Neurologic: easily aroused; good symmetric tone and strength; positive root and suck; symmetric normal reflexes

Newborn Hearing Screen (OAE):
Right Ear: Pass
Left Ear: Pass

Immunization History

Administered	Date(s) Administered
• Hepatitis B Vaccine (Peds/Adol 3-dose), IM	07/01/2015/

Anticipatory guidance performed: risks of co-sleeping, **breast feeding*****, formula feeding, bowel movements, bathing and cord care, **circumcision care*****, dental care, temperature, activity, smoking, stimulation, back to sleep, shaken baby syndrome, car seat safety, baby blues and when to call the doctor. Standard newborn care instructions and anticipatory guidance were provided to the mother prior to discharge.

Patient Active Problem List

Diagnosis
• Single liveborn infant delivered vaginally

Hospital Course: Baby GIRL/Test Test was born on 07/01/2015 at 0920 at Gestational Age: 39w6d via Delivery Method: VBAC, Spontaneous to a 24 y.o. G2P2002 . Peds {WAS/WAS NOT} called and no resuscitation was required. *** Baby did well overall and has fed, voided and stooled appropriately. Mom received anticipatory guidance on caring for her new baby. No other acute issues arose during this admission. Baby deemed medically stable for discharge. Baby to f/u with *** in 1-2 days.

- Baby's 24 hour CCHD Screen: SpO2: Pre-Ductal (Right Hand): 97 % and SpO2: Post-Ductal (Either Foot): 98 % Critical Congenital Heart Defect Score: Negative
- Baby's 40 hour bilirubin was ***
- Birth weight was 2990 g (6 lb 9.5 oz) (Filed from Delivery Summary)
- Discharge weight was 2830 g (6 lb 3.8 oz) (07/03 0125)
- Admission weight change of -5%

Studies pending: {None:"None"}
Special Follow-up Needed: {None:"None"}

Me, MD
07/03/2015 10:30 AM

Example 6: Discharge summary post-optimization

Blue highlight: Auto generated data. ***: Manual entry of data required. { }: Pick list. Epic codes are omitted.

Yo: Year old. GP: Gravida para. HIV: Human immunodeficiency virus, HBSAg: Hepatitis B antigen. GC: Gonorrhea. CT: Chlamydia. GBS: Group B streptococcus. RPR: Rapid plasma reagin. VBAC: Vaginal birth after cesarean section. ROM: Rupture of membranes. Peds: Pediatrics. GA: Gestational age. AGA: Appropriate for gestational age. SGA: Small for gestational age. LGA: Large for gestational age. OAE: Otoacoustic emissions. SpO2: Oxygen saturation. Bili: Bilirubin.
