# Supplementary material for: Saving Time for Patient Care by Optimizing Physician Note Templates: A Pilot Study
Source: Front Digit Health. 2022 Jan 13;3:772356. doi: 10.3389/fdgth.2021.772356 (PMC8792616; doi:10.3389/fdgth.2021.772356)
Supplement: Supplementary file 7 [file Data_Sheet_7.PDF]

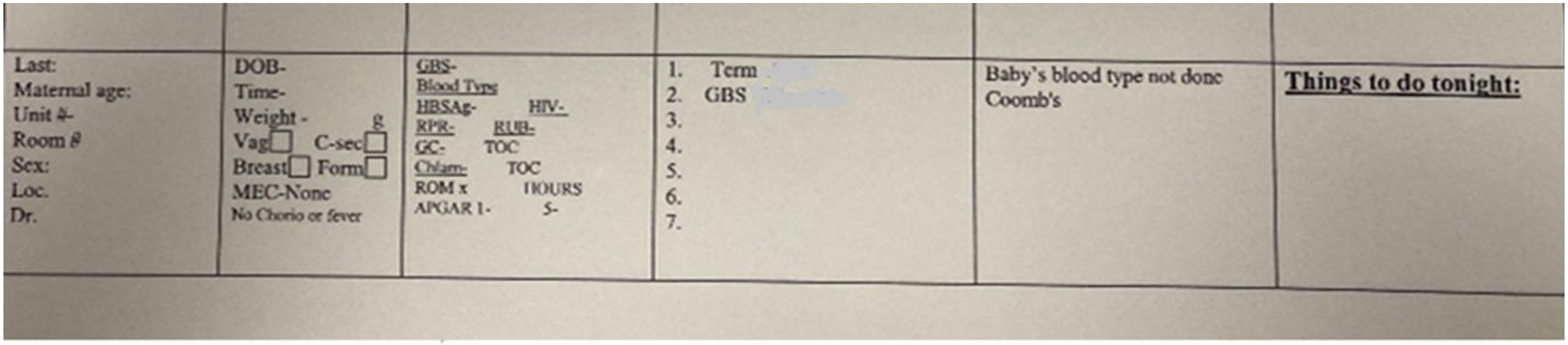

Last:<br>Maternal age:<br>Unit #-<br>Room #<br>Sex:<br>Loc.<br>Dr.	DOB-<br>Time-<br>Weight - g<br>Vag☐ C-sec☐<br>Breast☐ Form☐<br>MEC-None<br>No Chorio or fever	GBS-<br>Blood Type<br>HBSAg- HIV-<br>RPR- RUB-<br>GC- TOC<br>Chlam- TOC<br>ROM x HOURS<br>APGAR 1- 5-	1. Term [illegible]<br>2. GBS [illegible]<br>3.<br>4.<br>5.<br>6.<br>7.	Baby's blood type not done<br>Coomb's	**Things to do tonight:**

Example 7: Hand-off list pre-optimization

Loc: Location. DOB: Date of birth. Vag: Vaginal. C-sec: Cesarean section. Mec: Meconium. HIV: Human immunodeficiency virus, HBSAg: Hepatitis B antigen. GC: Gonorrhea. Chlam: Chlamydia. GBS: Group B streptococcus. RPR: Rapid plasma reagin. RUB: Rubella. Chorio: Chorioamnionitis. ROM: Rupture of membranes.
