# Supplementary material for: Saving Time for Patient Care by Optimizing Physician Note Templates: A Pilot Study
Source: Front Digit Health. 2022 Jan 13;3:772356. doi: 10.3389/fdgth.2021.772356 (PMC8792616; doi:10.3389/fdgth.2021.772356)
Supplement: Supplementary file 8 [file Data_Sheet_8.PDF]

Pt Info	Maternal Notes:	Maternal Labs:	Delivery Info	Pt Notes:	To Do:
**Name:** GIRL/Test Test<br>**MRN:** 22222222<br>**Sex:** female<br>**Room:** 310/310-01<br>**Dispo:** ***	**Age**: 24 y.o. GP: G2P1001<br>**PNC**: {Desc; adequate/inadequate}, *** visits at ***}<br>**PMH:** ***<br>**Meds**: ***	**HIV:** --/--/NON-REACTIVE/-- (06/24 1419)<br>**HBSAg:** NON-REACTIVE (06/24 1419)<br>**GC:**NOT DETECTED (06/29 1407)<br>**CT:** NOT DETECTED (06/29 1407)<br>**GBS:** positive<br>**RPR:** Negative (06/24 1419)<br>**Rubella:** 1.46 (06/23 1017)<br>**Blood:** A/Positive (06/24 1324)	**DOB:** 07/01/2015 **Time:** 9:20 AM<br>**Birth Weight:** 2.99 kg (6 lb 9.5 oz)<br>**Delivery Method:** **VBAC, Spontaneous**<br>**Peds Called?** {YES/NO}<br>**Resus:** None<br>**APGAR 1 min: 8 APGAR 5 min: 9**<br>**Fluid:** Clear [1]<br>**Chorio:** ***<br>**ROM (hrs):** 13h 52m	**GA**: 39 6/7 **TOB:** 9:20 AM<br>{AGA/SGA/LGA}<br>**Probs**: ***<br>**Phys Exam**: ***<br>**pH** 7.29* **BE:** 0.5<br>**Wt Change**: -5%	[ ] 40h Bili ***<br>[ ] v, [] s, [ ] FU, [ ] AG, [ ] OAE, [ ] SpO2, [ ] HepB, [ ] HC ***<br>[ ] 3rd Trimester Labs,<br>[ ] RR<br>Formula

Example 8: Hand-off list post-optimization

Blue highlight: Auto generated data. ***: Manual entry of data required. { }: Pick list. Epic codes are omitted.
Y.o.: Year old. Pt: Patient. Info: Information. Dispo: Disposition. PNC: Prenatal care. PMH: Past medical history. Meds: Medications. HIV: Human immunodeficiency virus, HBSAg: Hepatitis B antigen. GC: Gonorrhea. CT: Chlamydia. GBS: Group B streptococcus. RPR: Rapid plasma reagin. DOB: Date of birth. VBAC: Vaginal birth after cesarean section. Peds: Pediatrics, Resus: Resuscitation. Chorio: Chorioamnionitis. ROM: Rupture of membranes. GA: Gestational age. TOB: Time of birth. AGA: Appropriate for gestational age. SGA: Small for gestational age. LGA: Large for gestational age. Probs: Problems. Phys: Physical. BE: Base excess. Wt: Weight. Bili: bilirubin. V: Void. S: Stool. FU: Follow up. OAE: Otoacoustic emissions. SpO2: Oxygen saturation. Hep B: Hepatitis B vaccine. HC: Head circumference. RR: Red reflex.
